# Predicting range shifts of the giant pandas under future climate and land use scenarios

**DOI:** 10.1002/ece3.9298

**Published:** 2022-09-11

**Authors:** Zhenjun Liu, Xuzhe Zhao, Wei Wei, Mingsheng Hong, Hong Zhou, Junfeng Tang, Zejun Zhang

**Affiliations:** ^1^ Key Laboratory of Southwest China Wildlife Resources Conservation (Ministry of Education) China West Normal University Nanchong China; ^2^ Institute of Ecology, China West Normal University Nanchong China; ^3^ Liziping Giant Panda's Ecology and Conservation Observation and Research Station of Sichuan Province Nanchong China

**Keywords:** climate change, giant pandas, land use change, MaxEnt, range shifts

## Abstract

Understanding and predicting how species will respond to global environmental change (i.e., climate and land use change) is essential to efficiently inform conservation and management strategies for authorities and managers. Here, we assessed the combined effect of future climate and land use change on the potential range shifts of the giant pandas (*Ailuropoda melanoleuca*) in Sichuan Province, China. We used species distribution models (SDMs) to forecast range shifts of the giant pandas by the 2050s and 2070s under four combined climate and land use change scenarios. We also compared the differences in distributional changes of giant pandas among the five mountains in the study area. Our SDMs exhibited good model performance and were not overfitted, with a mean Boyce index of 0.960 ± 0.015 and a mean omission rate of 0.002 ± 0.003, and suggested that precipitation seasonality, annual mean temperature, the proportion of forest cover, and total annual precipitation are the most important factors in shaping the current distribution pattern of the giant pandas. Our projections of future species distribution also suggested a range expansion under an optimistic greenhouse gas emission, while suggesting a range contraction under a pessimistic greenhouse gas emission. Moreover, we found that there is considerable variation in the projected range change patterns among the five mountains in the study area. Especially, the suitable habitat of the giant panda is predicted to increase under all scenarios in the Minshan mountains, while is predicted to decrease under all scenarios in Daxiangling and Liangshan mountains, indicating the vulnerability of the giant pandas at low latitudes. Our findings highlight the importance of an integrated approach that combines climate and land use change to predict the future species distribution and the need for a spatial explicit consideration of the projected range change patterns of target species for guiding conservation and management strategies.

## Graphical abstract

Climate and land use changes affect the future distribution of the giant pandas.

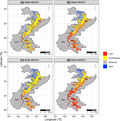

## 1 INTRODUCTION

The earth is undergoing serious biodiversity loss due to human‐induced global environment change (Pimm et al., 2014; Tittensor et al., 2014), of which climate change and land use change rank among the most important direct drivers of such trends (Di Febbraro et al., 2019; Sala et al., 2000). This threat is predicted to become more intense in the near future due to accelerating global warming and intensifying habitat fragmentation (Sirami et al., 2017; Titeux et al., 2017). To avoid the further loss of biodiversity, effective conservation programs are required to mitigate the negative impact of these factors (Barnosky et al., 2011). A foundational role of biodiversity conservation research is to understand how these factors might affect the future distribution of species so as to efficiently inform conservation and management strategies for authorities and managers (Coreau et al., 2009; Maggini et al., 2014).

A common practice for assessing the impacts of climate and land use change on species is to use species distribution models (SDMs) to project the species range shifts under future environmental conditions (Long et al., 2021; Marshall et al., 2018; Prestele et al., 2021; Schweiger et al., 2012). Most studies have projected the future species distribution under the combination of “dynamic” (i.e., change with time periods of projection) climate variables and “static” (i.e., remain unchanged) land use variables. However, due to the ongoing and continuing land use change, the static land use variables may not fully represent future species habitat suitability (Martin et al., 2013), which could potentially lead to unrealistic projections of future species distribution (Marshall et al., 2018; Sirami et al., 2017; Titeux et al., 2017). Moreover, the use of static land use has been criticized for neglecting the effect of land use change on the future distribution of a species (Barbet‐Massin et al., 2012). Therefore, an integrated approach that combing dynamic climate variables and dynamics land use variables is essential to project species' future distribution and assess the effect of land use change on species' distribution shifts. With the increase in available land use datasets at fine spatial scales under multiple future scenarios (Hurtt et al., 2011; Li et al., 2016), this type of integrated approach has been widely used to estimate species' future distribution changes for many taxonomic groups, such as plants (García‐Valdés et al., 2015; Zhang et al., 2017), insects (Marshall et al., 2018; Prestele et al., 2021), and mammals (Ma et al., 2021; Zamora‐Gutierrez et al., 2018).

As an iconic species and global symbol of conservation, the giant panda (*Ailuropoda melanoleuca*) has undergone pronounced human‐driven range contractions over the past 3000 years (Zhao et al., 2013), and now only lives in six isolated mountain ranges in Sichuan, Shaanxi, and Gansu provinces in southcentral China: the Qinling mountains of southeastern Shanxi province, the Minshan mountains of the southern Gansu province, the northwestern part of Sichuan province, and the Qionglai, Xiaoiangling, Daxiangling, and Liangshan mountains of southwestern Shanxi province. Over the past decades, China has established 67 panda nature reserves to protect this species from intensifying human activities and environmental changes (State Forestry Administration, 2021). Besides, two national‐level environmental protection projects: the Grain‐to‐Green Program (GTGP) and the Natural Forest Conversion Program (NFCP) were implemented to converse agricultural land with forested land, which also contributes to the recovery and improvement of panda habitats (Yang et al., 2017). Due to these conservation efforts, the giant pandas have recently been downlisted from Endangered to Vulnerable by the International Union for Conservation of Nature Red List (Swaisgood et al., 2016).

However, SDMs predicted that it will face serious risks from future climate change as a result of the dramatic loss of suitable habitats and intensifying habitat fragment (Li et al., 2015; Li et al., 2017; Shen et al., 2015; Songer et al., 2012; Wang et al., 2018). Moreover, previous studies have reported that giant pandas mainly inhabit primary forests (Hong et al., 2015; Zhang et al., 2011), and the knowledge regarding the effect of human‐induced land use patterns on the distribution and habitat selection of the giant panda at fine scales is well established (Bai et al., 2020; Wei et al., 2018; Yang et al., 2020). However, the effect of climate change and land use change, as well as their interactions, on a large‐scale distribution of the giant panda has rarely been explored. Tang et al. (2020) were the first to include dynamic land‐use changes on a large scale to assess the relative importance of climate change and land use change in determining the historical distribution patterns of the giant panda, finding that land use change could offset some of the negative effects of climate change on the giant pandas. Yet, to date, no studies have integrated climate change and land use change to project the future distribution of the giant panda at large scales.

To fill this gap, in this study we explored the combined effects of climate change and land use change on the future distribution changes in giant pandas. Our specific objectives are as follows: (1) identify the environmental requirements (i.e., ecological niche) of the giant pandas; (2) assess its habitat suitability under current environmental conditions; and (3) quantify changes in the area of suitable habitat under different future scenarios. To do so, we employed SDMs to project the habitat suitability of the giant pandas under current and future climate and land use conditions. Our study is the first attempt to investigate the combined effect of climate and land use change on the future distribution of the giant pandas, which could have important implications for future conservation strategies of this species.

## 2 MATERIALS AND METHODS

### 2.1 Study area and species occurrence data

This study was conducted in the five mountains (Minshan, Qionglai, Xiaoxiangling, Daxiangling, and Liangshan) of western Sichuan, China (102°29′36′′–102°52′24′′ E, 29°28′33′′–29°43′54′′ N)—home to about 75% of the giant pandas. The occurrence records of the giant pandas were provided by the Fourth National Giant Panda Survey (State Forestry Administration, 2021), which was carried out from 2011 to 2014. During this survey, panda presence was determined via signs (e.g., feces, fur, and signs of foraging; State Forestry Administration, 2021). In total, 3428 occurrence records for the giant pandas in our study area were obtained from this survey (Tang et al., 2020). To be consistent with the spatial resolution of our climate and land use variables (see below) and minimize the sampling bias effect in the occurrence records dataset, we created the same 1 × 1 km grid cells across the study area as the climatic and land use layers and all the occurrence records were overlaid onto these grid cells. After removing duplicate records within each gird cell, we obtained 2068 occurrence records to model ecological niches for the giant pandas.

### 2.2 Climate and land use data

The 19 bioclimatic variables (BIO1 – BIO19) for the current (1970–2000) and future (2050s [2041–2060] and 2070s [2061–2080]) time periods at a 1 km resolution were downloaded from the WorldClim 2.1 Database (Fick & Hijmans, 2017). For the future scenarios, we used the most recent climate simulations from the global circulation model (i.e., MRI‐ESM2‐0) that has been recommended for use in China (Rogelj et al., 2018) in two opposite greenhouse gas emissions scenarios: RCP 2.6 [low greenhouse gas emissions] and RCP 8.5 [high greenhouse gas emissions], respectively. The land use data were obtained from the Finer Resolution Observation and Monitoring‐Global Land Cover (FROM‐GLC) datasets, with a spatial resolution of 1 × 1 km (Li et al., 2016). The FROM‐GLC datasets include the proportion of 10 different land use types: (i) bare land, (ii) cropland, (iii) forest, (iv) grassland, (v) impervious, (vi) shrubland, (vii) snow/ice, (viii) urban green spaces, (ix) water, and (x) wetland. For consistency with climatic data, we extracted the above 10 variables for current (2010) and future (i.e., 2050 and 2070) under two RCP scenarios (i.e., RCP 2.6 and RCP 8.5). Finally, these 29 environmental variables were further subselected by checking for multicollinearity (VIF < 5) using the “vifstep” function in the “usdm” package (Version 1.1‐18; Naimi & Araújo, 2016), retaining the following eight variables: annual mean temperature (BIO1), temperature seasonality (BIO4), total annual precipitation (BIO12), precipitation seasonality (BIO15), the proportion of area covered by cropland (CL), forest (FL), shrubland (SL), and urban green spaces (UGSL) in grid cells (Table 1).

**TABLE 1:** The selected eight predictor variables used to model ecological niches for the giant pandas

Variable	Description	Units
BIO1	Annual mean temperature	°C
BIO4	Temperature seasonality	°C
BIO12	Total annual precipitation	mm
BIO15	Precipitation seasonality	
CL	The proportion of area covered by cropland	%
FL	The proportion of area covered by forest	%
SL	The proportion of area covered by shrubland	%
UGSL	The proportion of area covered by urban green spaces	%

### 2.3 Species distribution modeling

The maximum entropy algorithm (MaxEnt; Phillips et al., 2006) was used to make current and future projections of potentially suitable habitats for giant pandas. We chose MaxEnt because of its superior performance to model species distribution using presence‐only data compared to other algorithms (Elith et al., 2006). Moreover, recent studies suggested that the tuned MaxEnt models can perform comparably to ensemble SDMs (Hao et al., 2020; Low et al., 2021). To improve the performance of MaxEnt and avoid overfitting, following Jarvie et al. (2021), we ran 24 Maxent models based on all possible combinations of eight regularization multipliers (i.e., 0.5, 1, 1.5, 2, 2.5, 3, 3.5, and 4) and three feature class options (i.e., linear, linear/quadratic, and linear/quadratic/product). The performance of each model was evaluated using a non‐spatial fivefold cross‐validation. We used the corrected Akaike information criterion (AICc) to select the best‐performing model as this metric reflects both model goodness of fit and complexity (Muscarella et al., 2014). All the models were developed using the ENMeval package (Version 2.0.3; Kass et al., 2021) with the ENMevaluate function in the R platform (v. 4.1.3; http://cran.r‐project.org).

The performance of the optimal model was evaluated using the Boyce index (Boyce et al., 2002). We chose the Boyce index due to its superior performance compared to the two commonly used metrics, i.e., the area under the receiver operating characteristic curve (AUC) and the true skill statistic (TSS), for both the metrics may present a problem when presence‐only data are used (Jiménez‐Valverde, 2012; Leroy et al., 2018; Lobo et al., 2008). The Boyce index ranges from −1 to 1, with positive values indicating better model performance and negative values indicating performance no better than a random model (Hirzel et al., 2006). In addition, to assess whether the optimal model is overfitting, we also calculated the test point omission rate based on the minimum training presence value (OR_MTP_). This metric was threshold dependent and range from 0 (models that are not overfitted) to 1.0 (models that are overfitted; Peterson et al., 2011). The relative importance of the eight explanatory variables from the optimal model was determined by using the “permutation contribution,” a standard output of MaxEnt (Phillips et al., 2006). One habitat suitability was then produced using the optimal model for the current period and for each of the four different future scenarios (i.e., two RCP scenarios [RCP2.6 and RCP8.5] at two time periods [the 2050s and 2070s]), respectively. Finally, all these maps were converted into binary presence–absence maps by using the threshold the maximum sum of sensitivity and specificity, which has been frequently recommended (Liu et al., 2016).

### 2.4 Quantifying the distributional changes in giant pandas under future climate and land use change

For each future scenario, we assessed the distributional changes in giant pandas under future environmental conditions using the following three metrics: (i) the percentage of suitable habitat lost (i.e., number of grid cells that are suitable in the current period and become unsuitable in the future divided the number of currently suitable grid cells), (ii) the percentage of suitable habitat gained (i.e., number of grid cells that are unsuitable in the current period and become suitable in the future divided the number of currently suitable grid cells), and (iii) the net change ratios of suitable habitat (i.e., the percentage of suitable habitat gained minus the percentage of suitable habitat lost). To compare the differences in distributional changes of giant pandas among the five mountains, we also calculated the above three metrics for each of the five mountains.

## 3 RESULTS

### 3.1 Model performance and variable contributions

**FIGURE 1:**
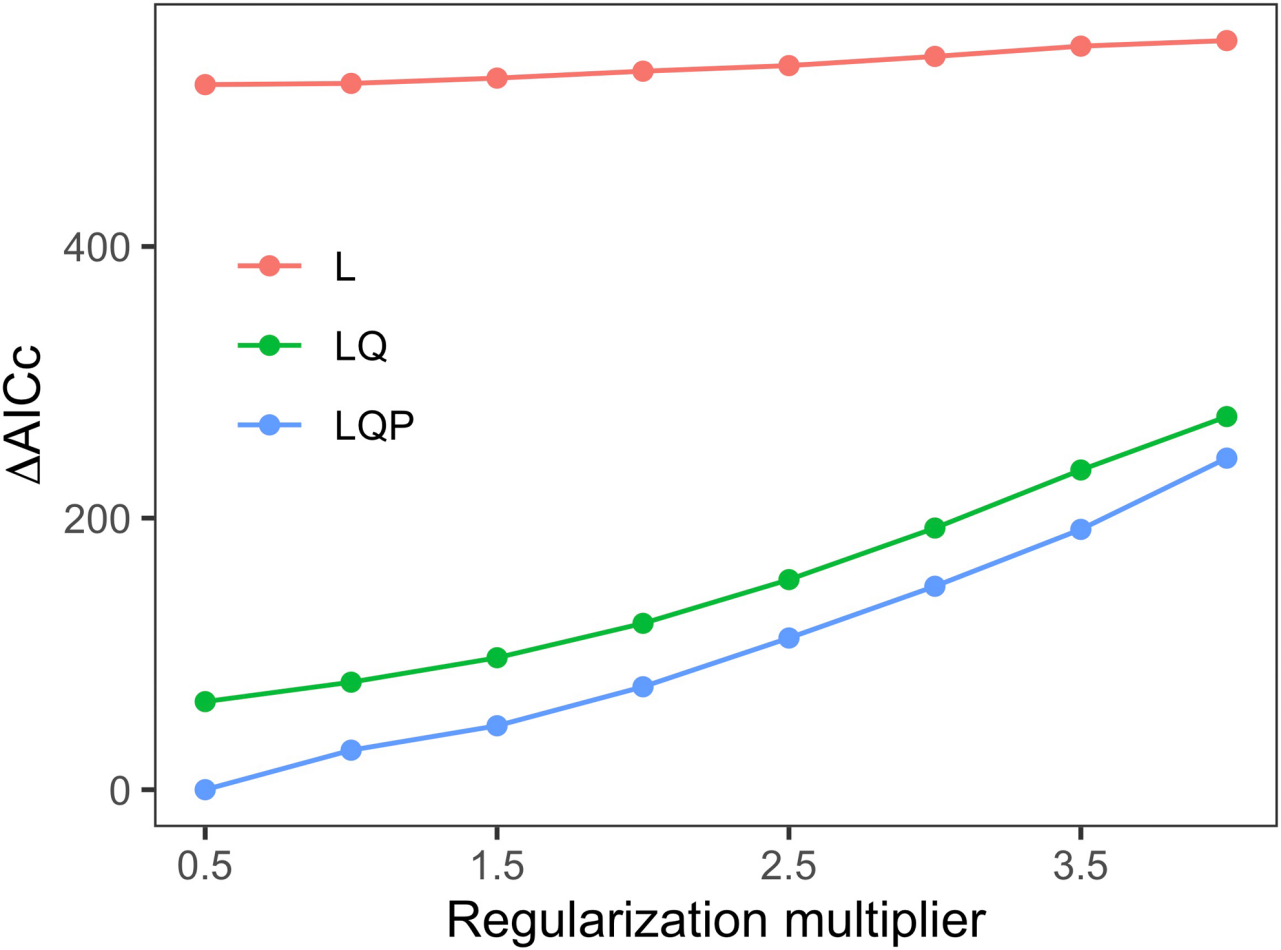
The ΔAICc (the corrected Akaike information criterion) values for the MaxEnt models under a range of feature combinations and regularization multipliers. The settings (regularization multiplier = 0.5 and feature combination = LQP) yielded the best‐performing model (ΔAICc = 0). L, linear feature; Q, quadratic feature; P, product feature.

**FIGURE 2:**
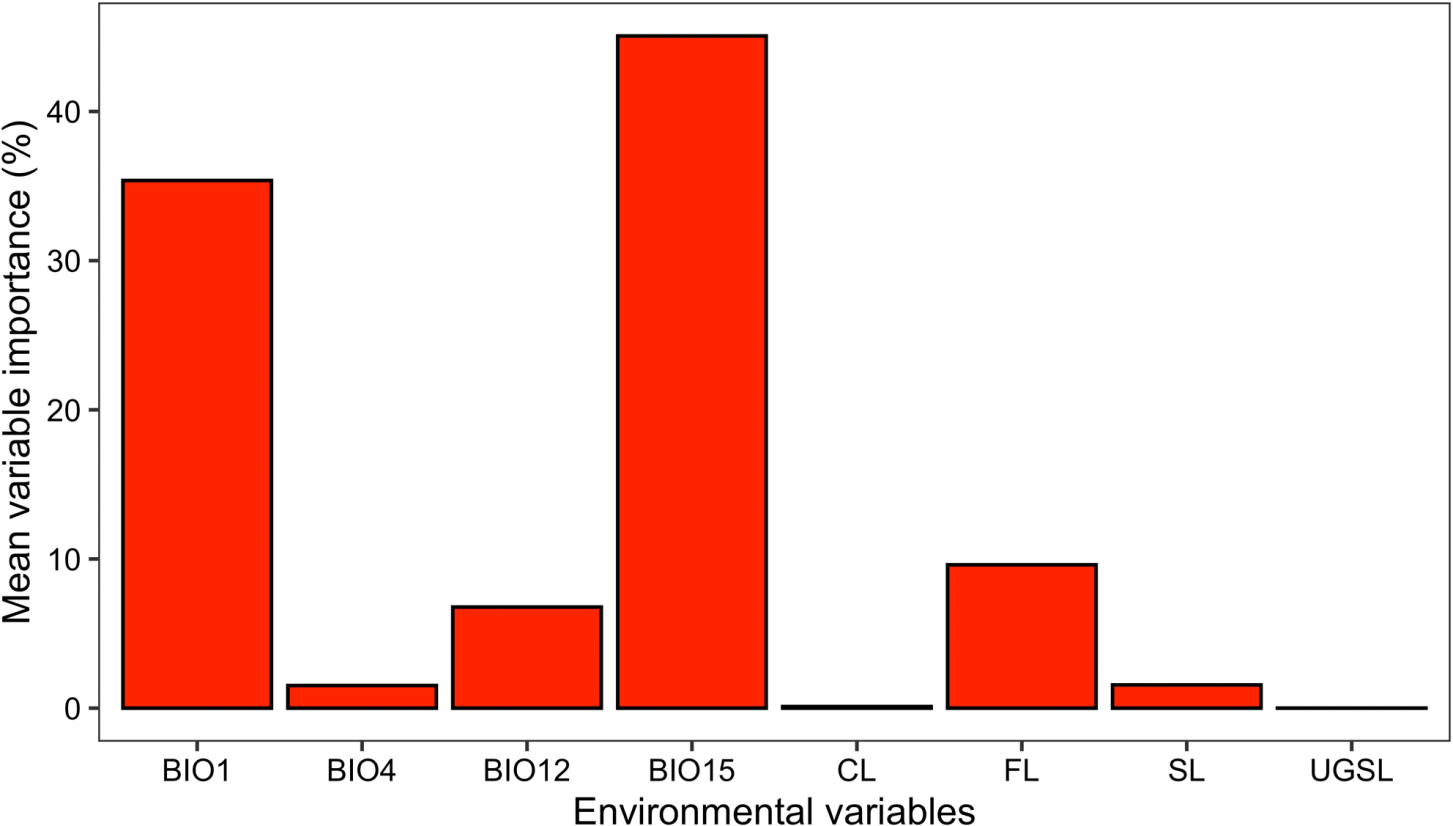
The mean variable importance of the eight selected environmental variables is included in the optimal MaxEnt model. BIO1: Annual mean temperature, BIO4: Temperature seasonality, BIO12: Total annual precipitation, BIO15: Precipitation seasonality, CL, FL, SL, and UGSL are the proportion of area covered by cropland, forest, shrubland, and urban green spaces in grid cells, respectively.

The settings (regularization multiplier = 0.5 and feature combination = linear/quadratic/product) yielded the optimal model (ΔAICc = 0) (Figure 1). The optimal models had an excellent predictive performance, with a mean Boyce index of 0.960 ± 0.015. Besides, the mean OR_MTP_ value is 0.002 ± 0.003, indicating that our models were not overfitted. Among the eight selected predictor variables, precipitation seasonality (BIO15) had the highest contribution to our model, followed by annual mean temperature (BIO1), the proportion of forest area (FL), and total annual precipitation (BIO12), while the remaining four variables contributed little to the distribution of the giant pandas (Figure 2).

### 3.2 Habitat suitability under current and future environmental conditions

The predicted potential suitable habitat area for the giant pandas in the study area under current and future environmental conditions is presented in Table 2. Under the current climate and land use conditions, the projected suitable habitat area for the giant pandas is 25,817 km^2^. Among the five mountains, Minshan mountains have the highest suitable area (11,706 km^2^), with a percentage of 45.34%, while Xiaoxiangling mountains have the lowest suitable area (431 km^2^), with a percentage of 1.67%. The potentially suitable habitat area in Qionglai, Daxiangling, and Liangshan mountains is 7600 km^2^ (29.44%), 1946 km^2^ (7.54%), and 4134 km^2^ (16.01%), respectively.

**TABLE 2:** Area of suitable habitat for giant pandas projected by the optimal MaxEnt models under current and future environmental conditions in the whole study area (Total) and in the five mountains: Minshan (MS), Qionglai (QL), Daxiangling (DXL), Xiaoxiangling (XXL), and Liangshan (LS) mountains.

Scenarios	Suitable habitat area (km^2^)					
	MS	QL	XXL	DXL	LS	Total
Current	11,706	7600	431	1946	4134	25,817
2050s RCP2.6	14,418	7707	432	1231	3115	26,903
2050s RCP8.5	12,953	4714	248	301	1118	19,334
2070s RCP2.6	16,731	8430	310	773	2081	28,325
2070s RCP8.5	12,803	2681	201	36	236	15,957

**FIGURE 3:**
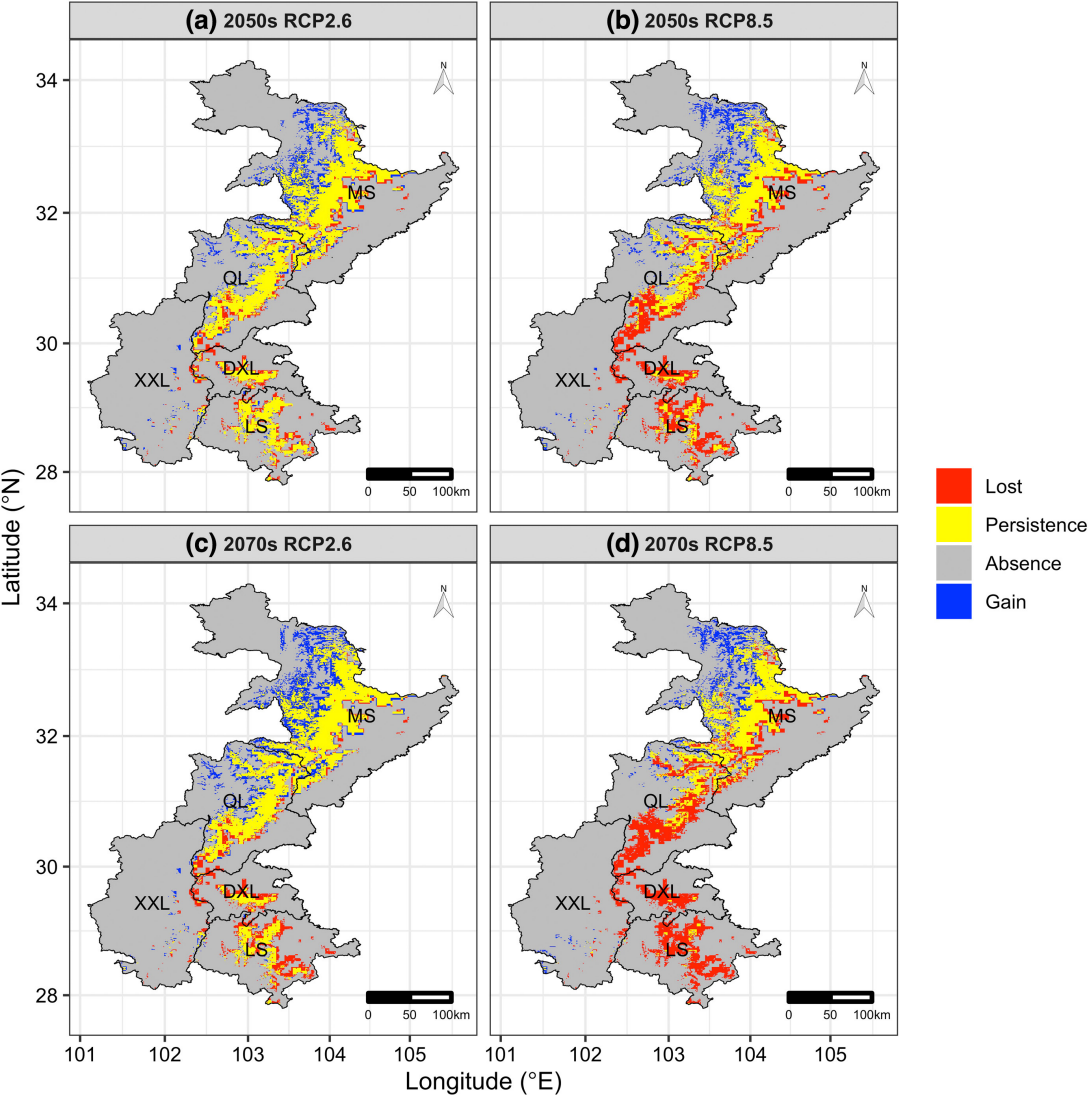
Predicted changes in suitable habitat for the giant pandas projected by the optimal MaxEnt model under different future scenarios: (a) under RCP 2.6 by the 2050s, (b) under RCP 8.5 by the 2050s, (c) under RCP 2.6 by the 2070s, and (d) under RCP 8.5 by the 2070s. MS, Minshan mountain; QL, Qionglai mountain; DXL, Daxiangling mountain; XXL, Xiaoxiangling mountain; LS, Liangshan mountain.

**FIGURE 4:**
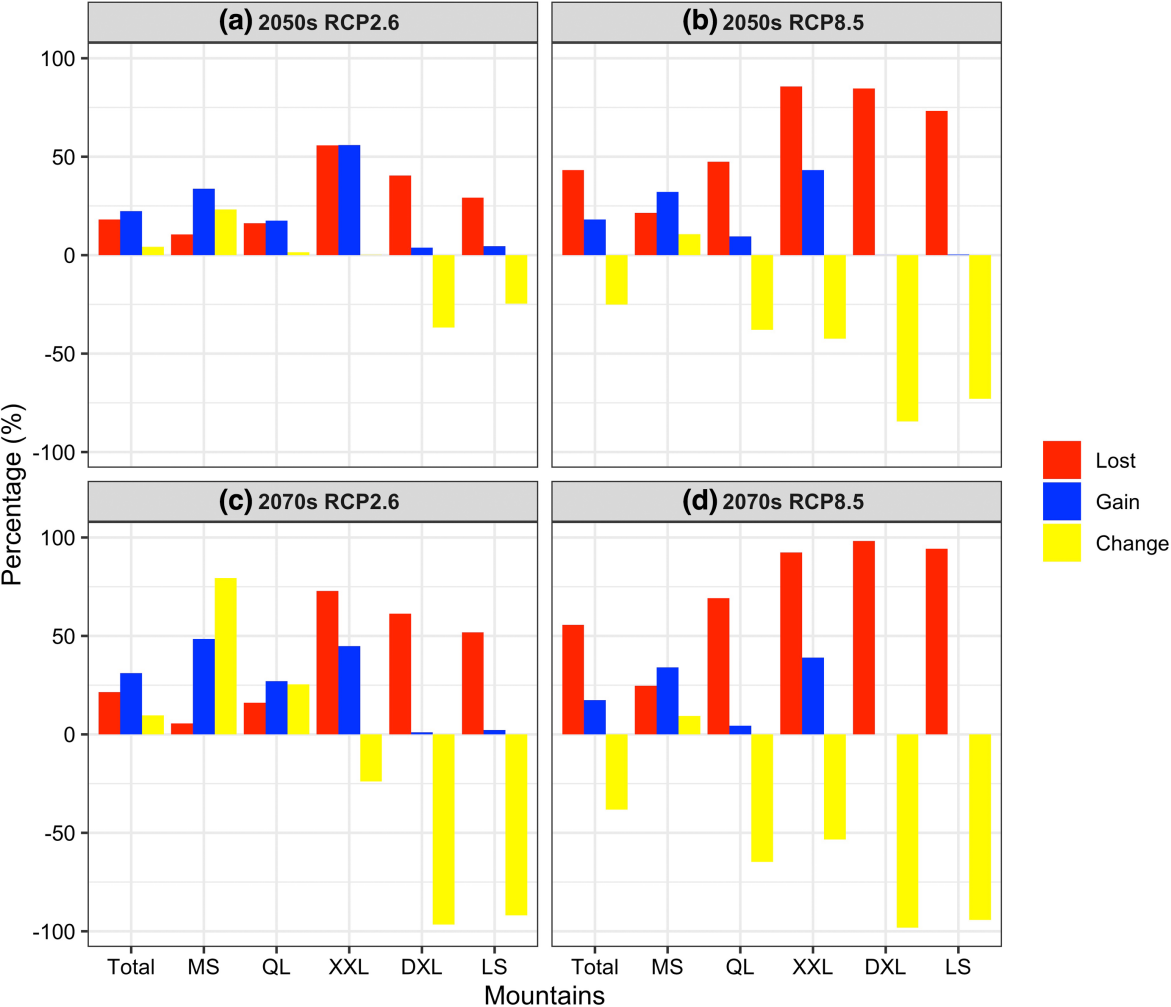
Percentage of suitable habitat lost (“Lost”), habitat gain (“Gain”), and net change ratios of suitable habitat change (“Change”) for the giant pandas predicted by the optimal MaxEnt model under future climate and land use change scenarios: (a) under RCP2.6 by 2050s; (b) under RCP8.5 by 2050s; (c) under RCP2.6 by 2070s; and (d) under RCP8.5 by 2070s.

Under future environmental conditions, the projected changes in habitat suitability of the giant pandas were extremely sensitive to RCP scenarios (Table 2, Figures 3 and 4). Specifically, under RCP 2.6, we predicted a net gain of 4.20% (1086 km^2^) in currently suitable habitats based on the projected loss of 18.17% (4690 km^2^) and a gain of 22.37% (5776 km^2^) by the 2050s, and a net gain of 9.72% (2508 km^2^) in currently suitable habitats based on the projected loss of 21.40% (5525 km^2^) and a gain of 31.12% (8033 km^2^) by the 2070s (Table 2, Figures 3 and 4). However, under RCP 8.5, we predicted a net loss of 25.11% (6483 km^2^) in currently suitable habitats based on the projected loss of 43.24% (11,164 km^2^) and a gain of 18.13% (4681 km^2^) by the 2050s, and a net loss of 38.19% (9860 km^2^) in currently suitable habitats based on the projected loss of 55.57% (14,346 km^2^) and a gain of 17.37% (4486 km^2^) by the 2070s (Table 2, Figures 3 and 4).

Besides, there is considerable variation in the projected range change patterns among the five mountains. Specifically, although the projected range change patterns of the giant pandas in Qionglai and Xiaoxiangling mountains were similar to the whole study area (Table 2, Figures 3 and 4), the projected range change patterns in the remaining three mountains exhibit two complete opposite trends, that is, the giant pandas will experience range expansion in Minshan mountains under all scenarios, with the degree of increase ranging from 9.37% (1097 km^2^) [under RCP 8.5 by the 2070s] to 79.42% (5025 km^2^) [under RCP 2.6 by the 2070s], while this species will experience severe range contraction in Daxiangling and Liangshan mountains under all scenarios, with the degree of decrease ranging from 24.65% (1019 km^2^) [under RCP 2.6 by the 2050s] to 94.29% (3898 km^2^) [under RCP 8.5 by the 2070s] in Liangshan mountains, and ranging from 36.74% (715 km^2^) [under RCP 2.6 by the 2050s] to 98.15% (1910 km^2^) [under RCP 8.5 by the 2070s] in Daxiangling mountains.

## 4 DISCUSSION

In this study, we provide the first empirical test of the combined impacts of climate and land use change on the future distribution changes of giant pandas. Projections suggest that the future changes in suitable habitats of the giant pandas largely depend on the greenhouse gas emission scenario, that is, the giant pandas will experience range expansion under RCP 2.6, but will experience range contraction under RCP 8.5 for the whole study area. Moreover, we found that there is considerable variation in the projected range change patterns among the five mountains, indicating the vulnerability of the giant pandas at low latitudes. These findings should inform the role of dynamics climate variables and dynamics land use variables on projected suitable habitats of the giant pandas and thus have important implications for guiding future conservation and management strategies.

It has been reported that changes in land‐use patterns have an important effect on the distribution and habitat selection of the giant pandas as these changes can have either a positive or negative effect on the habitat of the giant pandas, especially for the forest cover, a key natural resource for the giant pandas (Bai et al., 2020; Tang et al., 2020; Wei et al., 2018). Consistent with these studies, we found that land use variables are important drivers in determining the current distribution of the giant pandas in our study area. More importantly, among these land use variables, the proportion of the forest cover contributes the most to our optimal MaxEnt models. As with other species (e.g., Marshall et al., 2018), these findings highlight the importance of incorporating land use variables into the SDMs with only climate variables.

Previous studies have suggested that the giant pandas have shifted and will continue to shift toward high altitude and/or latitude in response to the ongoing climate change, which would lead to a dramatic loss of suitable habitat for the giant pandas ranging from 30% to 85% in the future (Li et al., 2015; Li et al., 2017; Shen et al., 2015; Songer et al., 2012; Wang et al., 2018). Consistent with these previous studies, our integrated models that combine climate and land use change also predicted that about 72% of the current suitable habitat of the giant pandas was distributed at high latitudes (i.e., Minshan and Qionglai mountains), and this proportion will further increase in the future. Nonetheless, compared to these previous studies, our integrated models predicted less range contraction, even range expansion under the low greenhouse gas emission scenario. For example, Li et al. (2015) predicted that at least 52.9% of the current suitable habitat of the giant pandas will be lost under future climate change, while our integrated models predicted that the suitable habitat area will decrease by at most 25% in the future. These findings suggest that conservation intervention to manage habitat could offset the negative effects of climate change on the future distribution of giant pandas to some extent.

Furthermore, our projections of the future distribution of the giant pandas indicate that Daxiangling and Liangshan mountains are the two most vulnerable mountains at low latitudes in the study area as the suitable habitat will decrease by more than 80% under RCP 8.5 for both of these two mountains. These findings are expected for several reasons. Firstly, the range of the current suitable habitat of the giant pandas in these two mountains is narrower than that in the mountains at high latitudes. Secondly, previous studies have suggested that the rate and magnitude of climate change is greater at low latitudes than at high latitudes (Trew & Maclean, 2021; Yuan et al., 2018). Lastly, the continued logging, livestock grazing, road construction, and other factors that lead to land use change have led and will continue to lead to serious habitat degradation for the giant pandas in Liangshan and Daxiangling mountains (State Forestry Administration, 2006, 2021). These rapid environmental changes, together with the narrow range of the giant pandas, could lead to a heavy loss of suitable habitat in these two mountains (Williams et al., 2007), which also highlights that the prior implementation of effective conservation programs in these two mountains is of great urgency and significance. Despite that, the Liangshan mountains have not been included in the recently established Giant Panda National Park (Xu et al., 2017).

Finally, a better understanding of how climatic and land‐use changes will interact to influence future species distributions provides a platform for more informed conservation management strategies. Compared to climatic variables, land use variables such as forest cover are more under the control of managers and decision‐makers at regional and local scales. Over the past decades, many conservation efforts, such as the establishment of protected areas and the implementation of NFCP and GTGP, have greatly contributed to the improvement of forest cover and accordingly contributed to the nascent recovery of the panda (Swaisgood et al., 2016, 2018). Similarly, our findings indicated that these conservation efforts would also have positive impacts on the future distribution of the giant pandas a the land use patterns would offset some of the negative effects of changing climate. Our findings also highlighted the need for a spatial explicit consideration of the projected range change patterns of target species when assessing the effect of climate and land use change on the future distribution of species as managers and decision‐makers should be informed when and where the target species is likely at risk. However, due to methodological limitations such as study area, data, and algorithms, our analyses might lead to uncertainties regarding the projected species distribution (Préau et al., 2022). Therefore, caution is warranted in generalizing our findings to local regions to avoid the risk of mismanagement and implement conservation strategies to mitigate climate change by adapting land use management (Oliver & Morecroft, 2014).

## AUTHOR CONTRIBUTIONS

**Zhenjun Liu:** Data curation (equal); formal analysis (equal); writing – original draft (equal). **Xuzhe Zhao:** Resources (equal). **Wei Wei:** Writing – review and editing (equal). **Mingsheng Hong:** Writing – review and editing (equal). **Hong Zhou:** Writing – review and editing (equal). **Junfeng Tang:** Supervision (equal); writing – review and editing (equal). **Zenjun Zhang:** Supervision (equal); writing – review and editing (equal).

## CONFLICT OF INTEREST

The authors declare no conflict of interest.

## Data Availability

Script to run examples of species distribution models in the programming language R, the occurrence records of the giant pandas, climate and land use data that used for modelling are openly available in Dryad at https://doi.org/10.5061/dryad.xd2547dk7.
